# *Fok I* and *Bsm I* gene polymorphism of vitamin D receptor and essential hypertension: a mechanistic link

**DOI:** 10.1186/s40885-022-00229-y

**Published:** 2023-02-15

**Authors:** Richa Awasthi, Priyanka Thapa Manger, Rajesh Kumar Khare

**Affiliations:** 1 Department of Biochemistry, Integral Institute of Medical Sciences and Research, Integral University, Lucknow, India; 2 Department of Medicine, Integral Institute of Medical Sciences and Research, Integral University, Lucknow, India

**Keywords:** Essential hypertension, Renin angiotensin system, Single nucleotide polymorphism, Calcitriol receptors, Cyclic adenosine monophosphate

## Abstract

The vitamin D receptor (*VDR*) gene serves as a good candidate gene for susceptibility to essential hypertension. The gene regulates the renin angiotensin system by influencing blood pressure regulation. Around 3% of the human genome is regulated by the vitamin D endocrine system. Several studies have reported mixed results with respect to relationship of *VDR* gene and hypertension. Observational evidence supports the concept that vitamin D plays a role in the pathogenesis of cardiovascular disease and arterial hypertension which is further supported by meta-analysis and case control studies reporting how *VDR* polymorphism leads to the onset and development of hypertension. In this review, we summarize the existing literature on the link between VDR and hypertension, including mechanistic studies, observational data, and clinical trials showing relationship of vitamin D level and hypertension with a focus on recent findings related to genetic studies that showed the relationship of *VDR* gene polymorphism with vitamin D level in hypertensive and normotensive groups. As a result, determining the association of *VDR* polymorphisms with essential hypertension is expected to aid in the risk assessment for the condition.

## Introduction

Hypertension is a global public health problem with high morbidity and mortality. According to the World Health Organization, one in every three adults worldwide has high blood pressure (BP), which accounts for approximately half of all deaths from cardiac disease and stroke [1]. Hypertension is expected to be 60% more prevalent worldwide by 2025, affecting 1.56 billion people [2]. It is predicted that over 90% of patients with high BP have primary hypertension [3]. Primary hypertension is a polygenic disease caused by the interaction of genetic and environmental factors [4]. Although primary hypertension cannot be cured, it can be managed with appropriate therapy (including lifestyle modifications and medications) [5].

Multiple factors that control BP contribute to developing primary hypertension. The two primary factors include problems in either hormonal (natriuretic hormone, renin-angiotensin-aldosterone system [RAAS]) mechanisms or disturbances in electrolytes (sodium, chloride, potassium). Natriuretic hormone causes an increase in sodium concentrations in cells, which causes BP to rise. The RAAS regulates sodium, potassium, and blood volume, which in turn regulates arterial BP. Angiotensin II and aldosterone are two hormones involved in the RAAS. Angiotensin II causes blood vessel narrowing, increases the release of chemicals that raise BP, and increases aldosterone production. Aldosterone maintains sodium and water levels in the blood. As a result, there is more blood, which puts more pressure on the heart and raises BP [3]. Meanwhile, evidence suggests that genetic factors may play an important role in the development of primary hypertension [1]. It has been estimated that the heritability of hypertension ranges from 31 to 68%. Genome-wide association studies (GWAS) in several multinational cohorts have identified a large number of single nucleotide polymorphisms (SNPs) associated with hypertension [6].

Prospective studies showed that subjects with low vitamin D level was three times more likely to have hypertension than those with high vitamin D concentrations and also suggested that vitamin D receptor (*VDR*) polymorphism is associated with hypertension [7]. Since the *VDR* is widely distributed in vascular endothelial cells, vascular smooth muscle cells and cardiomyocytes, the role of vitamin D and *VDR* in hypertension has received extensive attention [1].

## Vitamin D and vitamin D receptor

Vitamin D is a group of fat-soluble molecules, and the most important of which are vitamin D_2_ (ergocalciferol) and vitamin D_3_ (cholecalciferol). A large amount of vitamin D is converted to vitamin D_3_ from 7-dehydrocholesterol after exposure to ultraviolet B in the skin. Furthermore, just 10 to 20% of vitamin D comes from the diet as vitamin D_2_ or vitamin D_3_ [8]. Foods rich in vitamin D are mainly cod-liver oil, fish, animal liver, eggs, and fortified milk [1].

After entering the body, vitamin D is hydroxylated in the liver to 25-hydroxyvitamin D (25(OH)D, calcidiol), which is the major circulating form of vitamin D and is used to assess overall vitamin D status. The kidney converts 25(OH)D to its bioactive form, 1,25-dihydroxyvitamin D (1,25(OH)_2_D_3_, calcitriol), which has much stronger specificity with VDR and is responsible for the majority of vitamin D metabolic actions [9].

**Fig. 1:**
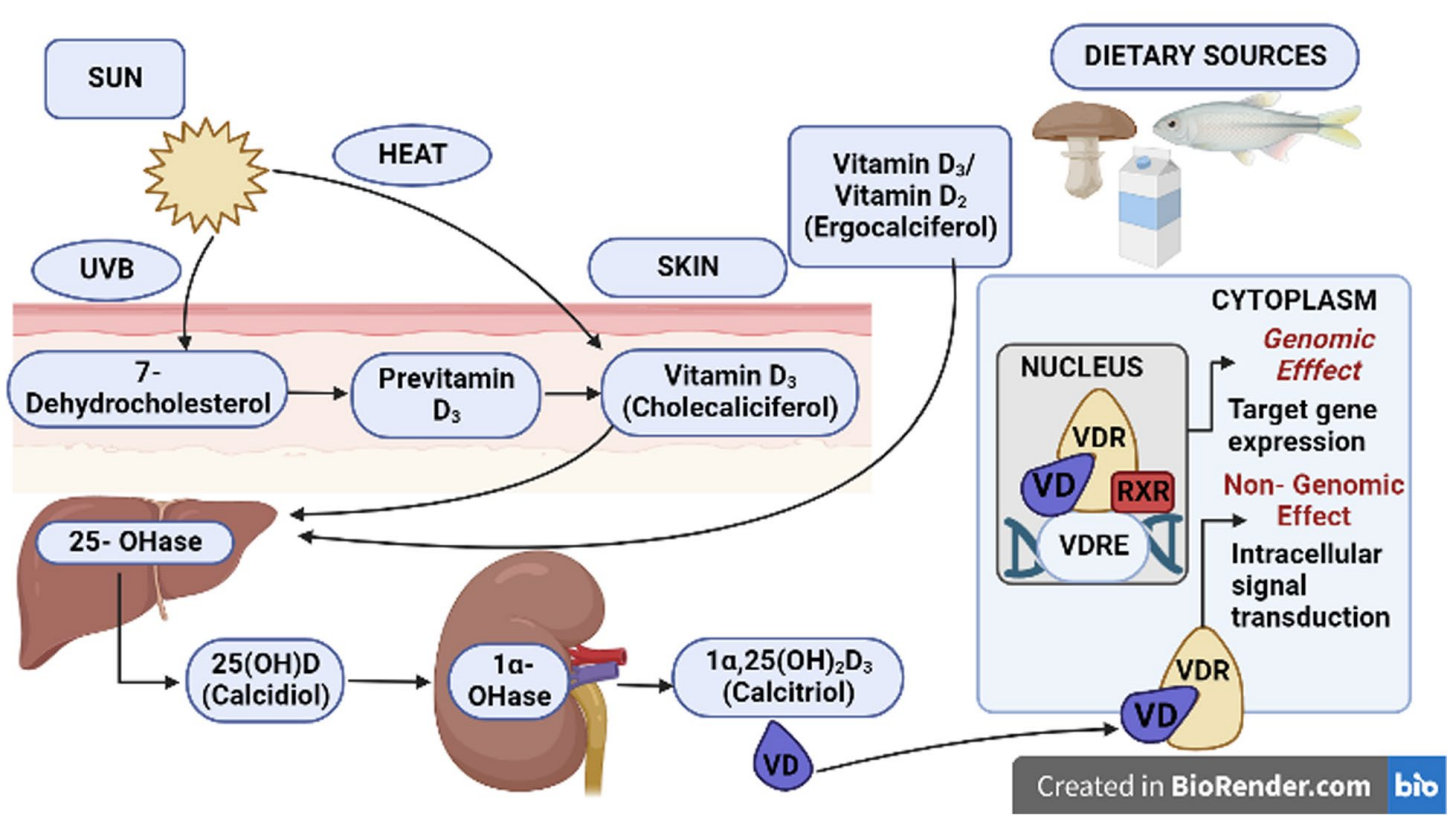
Mechanism of action of vitamin D (VD) by binding through its receptor. UVB, ultraviolet B; OHase, hydroxylase; 25(OH)D, 25-hydroxyvitamin D; VDR, vitamin D receptor; RXR: retinoid X receptor; VDRE, vitamin D responsive elements. Created with BioRender (Toronto, Canada; https://biorender.com/)

VDR is a nuclear receptor protein that has a high affinity and specificity for binding to vitamin D_3_ (a form of vitamin D). When occupied by vitamin D_3_, *VDR* is phosphorylated, resulting in a change in surface conformation. The activated *VDR* then binds to the retinoid X receptor (*RXR*) to form a heterodimer that binds to vitamin D-responsive elements in the gene promoter region [10]. Activated *VDR/RXR* regulates the transcription of genes encoding proteins that perform traditional and nontraditional D_3_ functions such as maintaining musculoskeletal health, calcium homeostasis, normal BP, and cardiovascular function by recruiting complexes of either coactivators or corepressors [11]. Surprisingly, liganded 1,25(OH)_2_D_3_-*VDR* can interact directly with cyclic adenosine monophosphate (cAMP) dependent response element (CRE) binding protein (CREB), blunting its binding to CRE. These activities appear to be carried out in the absence of liganded-*VDR* heterodimerization with *RXR*. As a result, the expression of specific target genes will be regulated, facilitating the synthesis of vitamin D-regulated proteins (Fig. 1) [12, 13].

Human *VDR* is encoded by a single gene on chromosome 12 at 12q12–14. By modulating the expression of target genes, this protein mediates the pleiotropic effects of 1,25(OH)_2_D_3_ [14]. 1,25(OH)_2_D_3_ is a negative endocrine-regulating hormone in the RAAS that acts by inhibiting renin messenger RNA (mRNA) expression regardless of calcium metabolism, which is involved in bone function. Biological activities of vitamin D are mediated by binding to the *VDR*, where SNPs can cause changes in arterial BP and contribute to the onset of hypertension [15]. In recent decades, it has been found *VDR* exists in almost all human cells and modulates about 3% of human genes by activating a transcription of target gene. So, more and more attention has been paid to the role of vitamin D in non-skeletal diseases, including diabetes mellitus, autoimmune disease, hypertension, and cardiovascular disease (CVD).

## Mechanistic links of vitamin D receptor with hypertension

The genes that control vitamin D levels are thought to be responsible for 30 to 50% of BP fluctuations [13]. Identifying the genes that control vitamin D levels in essential hypertension may thus provide a better factor in determining the disease molecular pathogenesis.

The RAAS is critical in the physiologic regulation of sodium and potassium balance, intravascular volume, and BP [16]. It is now well established that excessive RAAS activity increases the risk of cardiovascular disease, which can be reduced by inhibiting or blocking the RAAS [17].

## Renin-angiotensin-aldosterone system

Increased activation of the RAAS, which is a key regulator of electrolyte and volume homeostasis, contributes to the development of arterial hypertension. Renin is primarily produced by the kidney’s juxtaglomerular cells and stimulates the production of angiotensin II and aldosterone, both of which increase BP directly through vasoconstriction and indirectly through salt and water retention and other mechanisms [18].

Low vitamin D status has been linked to clinical outcomes that have previously been linked to excess RAAS activity, such as hypertension, inflammation, and CVD [19]. Animal studies and human genetic association studies have provided mechanistic support for these observations; however, conflicting data exist, and large-scale studies are needed to confirm the effect of vitamin D therapy on the RAAS and RAAS-mediated clinical outcomes.

Animal studies have shown that the 1,25(OH)_2_D_3_-*VDR* complex negatively regulates renin expression and that this vitamin D-induced reduction in RAAS activity can prevent adverse vascular outcomes to the same extent as pharmacologic angiotensin receptor antagonism [20]. In *VDR* and 1α-hydroxylase knockout mice, inappropriate, increased RAAS activation has been reported. Importantly, *VDR* and 1α-hydroxylase knockout mice developed arterial hypertension and myocardial hypertrophy, even after calcium homeostasis was restored; however, blocking the RAAS with angiotensin-converting enzyme inhibitors normalized BP and cardiac abnormalities [21, 22]. Furthermore, in 1α-hydroxylase knockout mice, increased RAAS activation, arterial hypertension, and myocardial abnormalities could be successfully treated with 1,25(OH)_2_D_3_ [22].

Human studies have supported this theory, demonstrating that low circulating vitamin D concentration are associated with higher plasma renin activity and angiotensin II concentrations [23, 24], and that vitamin D deficiency is associated with higher RAAS activity, which can be reduced with vitamin D_3_ therapy intervention [20, 25]. Extrapolating from these findings, vitamin D therapy may help to lower RAAS activity and improve complications associated with excess RAAS activity, such as hypertension, insulin resistance, and nephropathy [26].

## Cyclic adenosine monophosphate protein kinase a signaling pathway

cAMP has long been recognized as a significant intracellular signal that stimulates renin production in juxtaglomerular cells. It is well established that cAMP signals through CRE located in target gene promoters, which interact in homodimeric or heterodimeric forms with members of the activating transcriptional factor/CREB/CRE modulator (CREM)/basic leucine zipper domain transcription factor family. Intracellular cAMP is synthesized from ATP by adenylate cyclase, which is activated by membrane receptors; cAMP binds to the regulatory subunit of protein kinase A, releasing the catalytic subunit, which enters the nucleus and phosphorylates CREB at serine 133 or CREM at serine 117, resulting in the recruitment of ubiquitous coactivators cyclic binding protein and p300 to promote gene transcription [27].

**Fig. 2:**
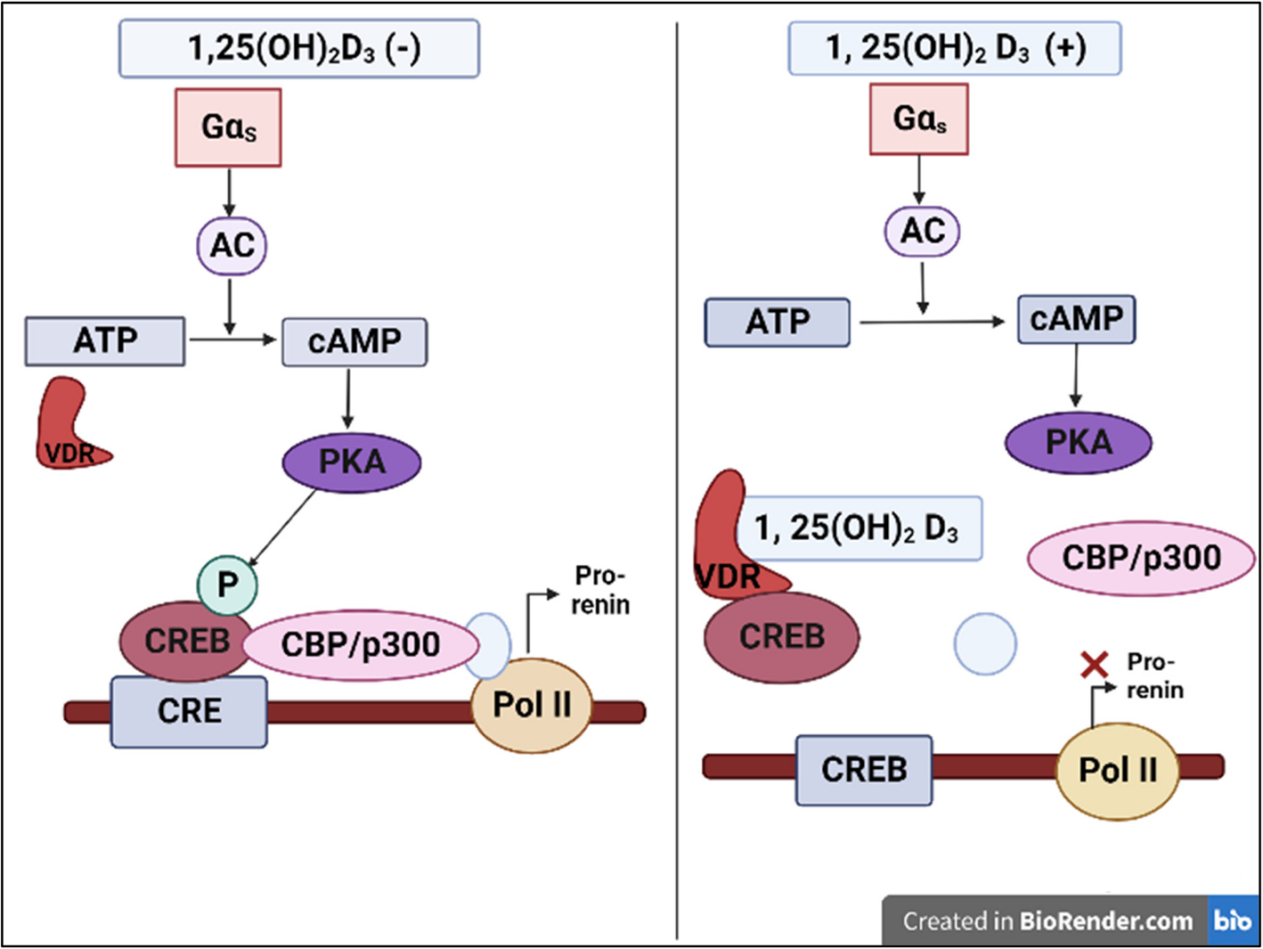
Proposed mechanism for action of vitamin D on renin expression. 1,25(OH)_2_D_3_, 1,25-dihydroxyvitamin D; Gα_s_, G_s_ protein α subunit; AC, adenylate cyclase; cAMP, cyclic adenosine monophosphate; VDR, vitamin D receptor; PKA, protein kinase A; P, phosphate; CREB, cyclic adenosine monophosphate dependent response element binding protein; CRE, cyclic adenosine monophosphate response element; CBP, cyclic adenosine monophosphate binding protein; Pol, polymerase. Created with BioRender (Toronto, Canada; https://biorender.com/). Adapted from Legarth et al. [12] according to the Creative Commons Attribution License

The discovery that liganded *VDR* suppresses renin expression in the presence of 1,25(OH)_2_D_3_ by binding to the transcription factor CREB sheds light on the molecular effects of vitamin D on the RAAS. CREB can no longer stimulate renin transcription by binding to CRE in the renin gene promoter region, so renin transcription is inhibited [27]. In patients with arterial hypertension, renin activity was found to be inversely related to 1,25(OH)_2_D_3_ levels [28]. Importantly, reduced renin and angiotensin II levels were observed in several, but not all, studies that investigated RAAS activity after treatment with 1,25(OH)_2_D_3_ (Fig. 2) [12, 29]. As a result, more research is needed to determine the clinical significance of vitamin D.

Increasing molecular and clinical evidence suggests that vitamin D deficiency contributes to insulin resistance, which is thought to play a role in the pathogenesis of arterial hypertension. Furthermore, the renoprotective effects of vitamin, such as decreased podocyte loss and hypertrophy or suppression of mesangial cell proliferation, may prevent the development of arterial hypertension in the course of renal failure [30].

## Vitamin D receptor polymorphisms

All the mechanisms discussed play a role in developing hypertension as well as polymorphism in the *VDR* gene which binds to high-affinity receptors mediates the biological activities of 1,25(OH)_2_D_3_, and polymorphisms in the gene encoding the *VDR* appear to predispose the onset of hypertension [31, 32].

**Fig. 3:**
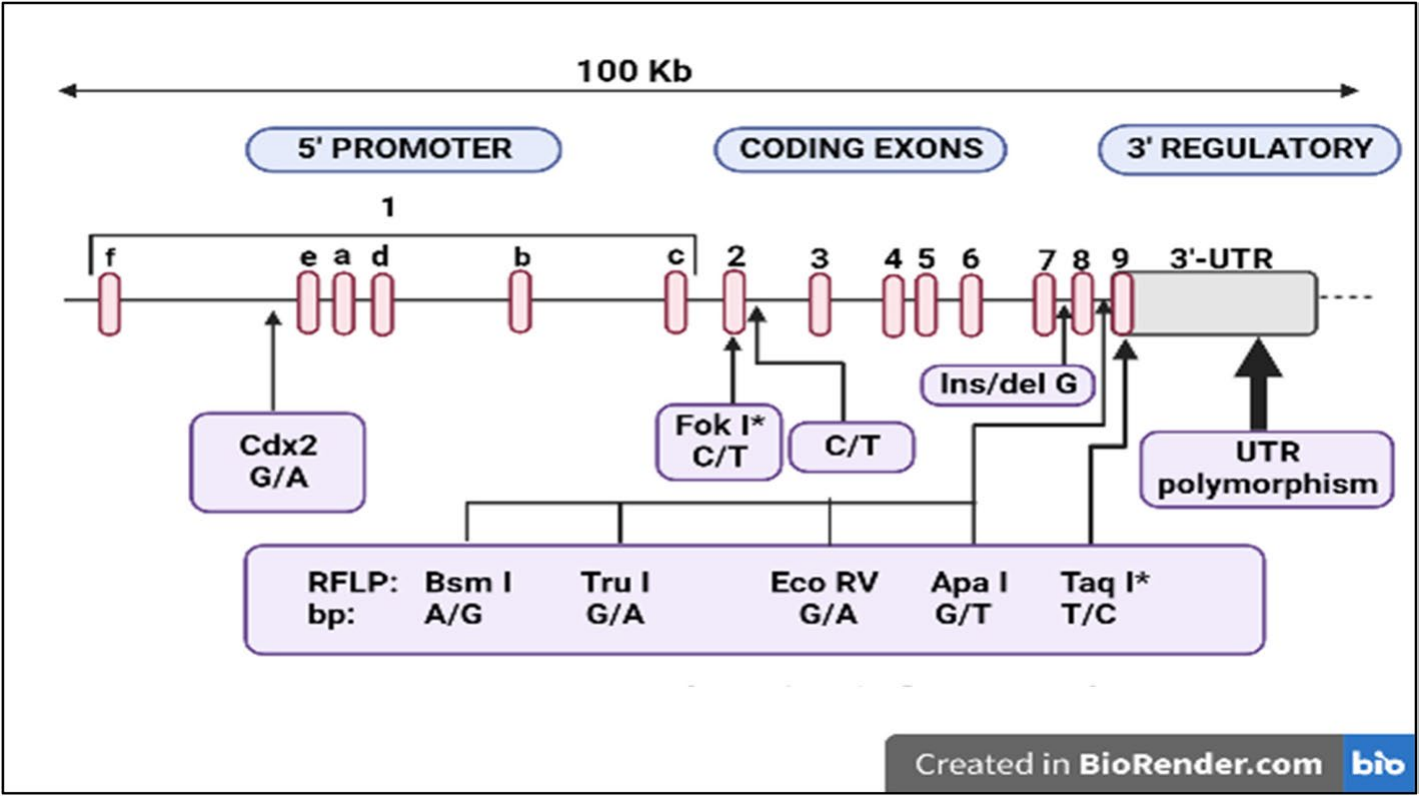
Structure of vitamin D receptor gene and position of known polymorphisms. Asterisks indicate that these polymorphisms are in the coding sequence. UTR, untranslated region. Created with BioRender (Toronto, Canada; https://biorender.com/). Adapted from Uitterlinden et al. [15] with permission from Elseiver

The expression and nuclear activation of the *VDR* are required for vitamin D effects. Several genetic variations in the *VDR* have been discovered. DNA sequence variations, which occur frequently in the population, are referred to as “polymorphisms” and can have biological effects. To see whether there is a linkage between *VDR* polymorphisms and diseases, epidemiological studies are performed. In these studies, the presence of a variation of the gene is studied in a case and control group [33]. Since the discovery of *VDR* polymorphism a number of papers have been published studying *VDR* gene and the position of different SNPs and their role in various diseases (Table 1, Fig. 3) [15, 34–41].

**Table 1:** Association studies of known SNPs of *VDR* gene with chronic disease

Study	Year	Country	Type of study	SNP	Disease	Outcome
Liu et al. [34]	2021	China	Meta-analysis	*Bsm I, Apa I, Taq I, Fok I*	GDM	*Taq I* and *Apa I* associated with risk of GDM. No association found between *Bsm I* and *Taq I* with the risk of GDM.
Tizaoui et al. [35]	2015	Tunisia	Meta-analysis (PRISMA)	*Taq I, Bsm I, Fok I*	RA	*Taq I* and *Fok I VDR* polymorphisms significantly associated with RA risk. *Bsm I,* marginal association seen with RA.
Magiełda-Stola et al. [36]	2021	Poland	Case control	*Bsm I, Apa I, Fok I, Taq I*	Preeclampsia	*Bsm I* is significantly found more frequent in preeclamptic women. *Taq I/Apa I/Bsm I* was significantly associated with higher systolic and diastolic blood pressure. *Bsm I* polymorphism is closely associated with a higher predisposition to hypertension.
Nam et al. [37]	2021	Republic of Korea	Cross sectional	*Bsm I, Apa I*	Obesity DM	*Bsm I* and *Apa I* were highly associated with obesity. Both SNPs were not associated with DM.
Abouzid et al. [38]	2021	Poland	Preliminary	*Bsm I, Apa I, Fok I, Taq I*	CVD	*Apa I, Tag I,* and *Bsm I* was found to be associated with an increased risk of obesity. *Fok I* was associated with a higher incidence of heart failure and hypertension. *Bsm I* was found to be associated with lower risk of CVD.
Eweida et al. [39]	2021	Egypt	Case control	*Bsm I, Fok I*	CVD	Both SNPs are associated with risk of CVD in Egyptian patients with or without diabetes.
Caccamo et al. [40]	2020	Italy	Case control	*Fok I, Bsm I*	GH	Significant association was found between *Fok I* and *Bsm I* with GH.
Aristizabal-Pachon et al. [41]	2022	Colombia	Case control	*Fok I, Taq I*	Melanoma cancer	*Fok I* polymorphism was associated with melonma cancer risk. *Taq I* polymorphism was associated with a protective effect against this cancer.

*SNP* single nucleotide polymorphism, *VDR* vitamin D receptor, *GDM* gestational diabetes mellitus, *RA* rheumatoid arthritis, *DM* diabetes mellitus, *CVD* cardiovascular disease, *GH* gestational hypertension

Among the many described SNPs of *VDR*, two diallelic polymorphisms have been reported to affect *VDR* molecular signaling, namely *Fok I* (C > T, rs2228750) within the first coding exon, and *Bsm I* (A > G, rs1544410) lying in the last intron [33]. The *VDR Fok I*-F mutated allele has been shown to increase *VDR* protein transcriptional activity, while the *Bsm I-B* mutated allele affects *VDR* mRNA stability leading to a reduction of VDR protein amount in tissues. It has been shown that these genetic changes in the *VDR* can significantly reduce the effectiveness of vitamin D action [33]; thus, contributing to the development of several cardiovascular diseases, in particular, the *Bsm I-B* allele has been strongly associated with hypertension [42, 43]. The purpose of this review is to summarize the vast amount of information regarding VDR polymorphisms and essential hypertension and discuss its possible role as diagnostic tools.

## *Fok I* polymorphism and essential hypertension

**Fig. 4:**
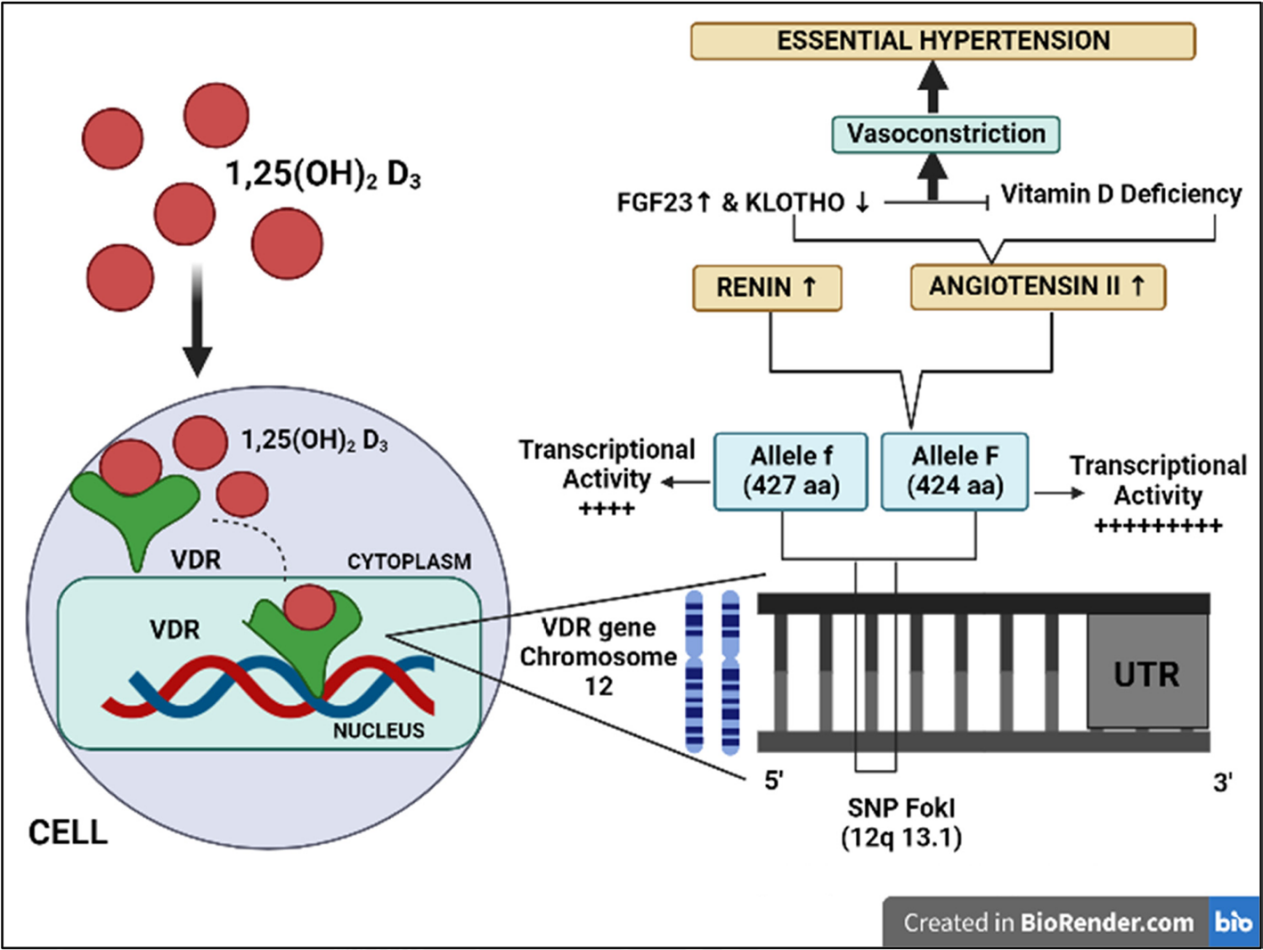
Proposed mechanism of vitamin D receptor (VDR; *Fok I*) polymorphism in susceptibility to essential hypertension. 1,25(OH)_2_D_3_, 1,25-dihydroxyvitamin D; SNP, single nucleotide polymorphism; UTR, untranslated region. Adapted from Nunes et al. [47] with permission from Oxford University Press

One of the most studied SNP of the *VDR* gene is *Fok I* (rs2228570). This polymorphism, which consisted of a T to C change in exon 2, was described in the early 1990s. Because the change occurs within a start codon (ATG), when the C variant is present, an alternate start site is used, resulting in a protein of a different size [33]. *Fok I* polymorphism can generate truncated proteins and is associated with increased risk for hypertension [15, 44, 45]. *Fok I* polymorphism is caused by a thymine-to-cytosine transition, which leads to a translational frameshift characterized by an extension of the open reading frame to the next initiation codon (ATG), resulting in the synthesis of a truncated 424-amino acid protein. In the 427-amino acid protein, ATG-encoded methionine (M1 form) was present in the f allele, whereas ACG-encoded methionine (M4 form) was present in the F allele [46]. The truncated protein in individuals with the FF genotype is thought to promote the development of essential hypertension by increasing the production of renin and angiotensin II (Fig. 4) [47, 48]. The transcriptional activity of the truncated protein is suggested to be higher than that of the full-length protein. Moreover, the increased responsiveness of the truncated protein to 1,25(OH)_2_D_3_ might alter the function of *VDR* and vitamin D in cells and tissues [49]. Low levels of 25(OH)D combined with *Fok I* polymorphism have been associated with increased plasmatic renin activity [12]. This suggests that 1,25(OH)_2_D_3_ can downregulate renin expression in humans, and increase cardiovascular and metabolic disease risk [50].

Regarding the role of SNP *Fok I* in other diseases, Liu et al. [51] analyzed its association with psoriasis but the results were not significant for Caucasians and East Asians. In contrast, Alizadeh et al. [52] found no significant association between SNP *Fok I* and coronary artery disease (CAD) based on a general analysis in Caucasians and East Asians. Recently, Shi et al. [53] observed no relation between this SNP and susceptibility to polycystic ovary syndrome. However, Liu et al. [54] showed that *Fok I* is a susceptibility factor for ovarian cancer. Further, Zhao et al. [55] found an increased risk of intervertebral disc degeneration in Hispanics and Asians with the f allele and Lu et al. [56] suggested that *Fok I* protects against CAD. Cao et al. [57] found that homozygosis is associated with an increased risk of tuberculosis, particularly in East and Southeast Asia, and Jiao et al. [58] observed that the F allele is associated with a decreased risk of diabetic retinopathy in Chinese subjects. However, Tizaoui et al. [35] in their study reported that *Fok I VDR* polymorphisms is associated with rheumatoid arthritis risk. Liu et al. [34] reported that *Fok I* (rs2228570) polymorphisms increase susceptibility to gestational diabetes mellitus in Asian and African population. Another study conducted by Caccamo et al. [40] suggested an association between gestational hypertension and the *VDR FF* haplotype. In accordance with previous study Abouzid et al. [38] reported that *Fok I* SNP TT genotype was associated with a higher incidence of heart failure and hypertension.

In the mid-2000s, an in vitro study demonstrated that the transcriptional activity of *VDR* with *Fok I*-F SNP was lower than that of TFIIB factor (an RNA polymerase II-specific transcript) with *Fok I*-F SNP. Thus, the *Fok I* polymorphism appears to be functional, and the 424 aa *VDR* variant appears to be slightly more active than the 427 aa variants in terms of transactivation capacity as a transcription factor. Some promoter regions of vitamin D target genes may be more sensitive to this *VDR* genotype-dependent difference in activity than others [59]. Due to the importance of the association between hypertension and *Fok I* polymorphism, we performed a review to find out whether this SNP of *VDR* gene plays a protective role in hypertension or should be considered as a risk factor for the onset of the disease.

## *Bsm I* polymorphism and essential hypertension

**Fig. 5:**
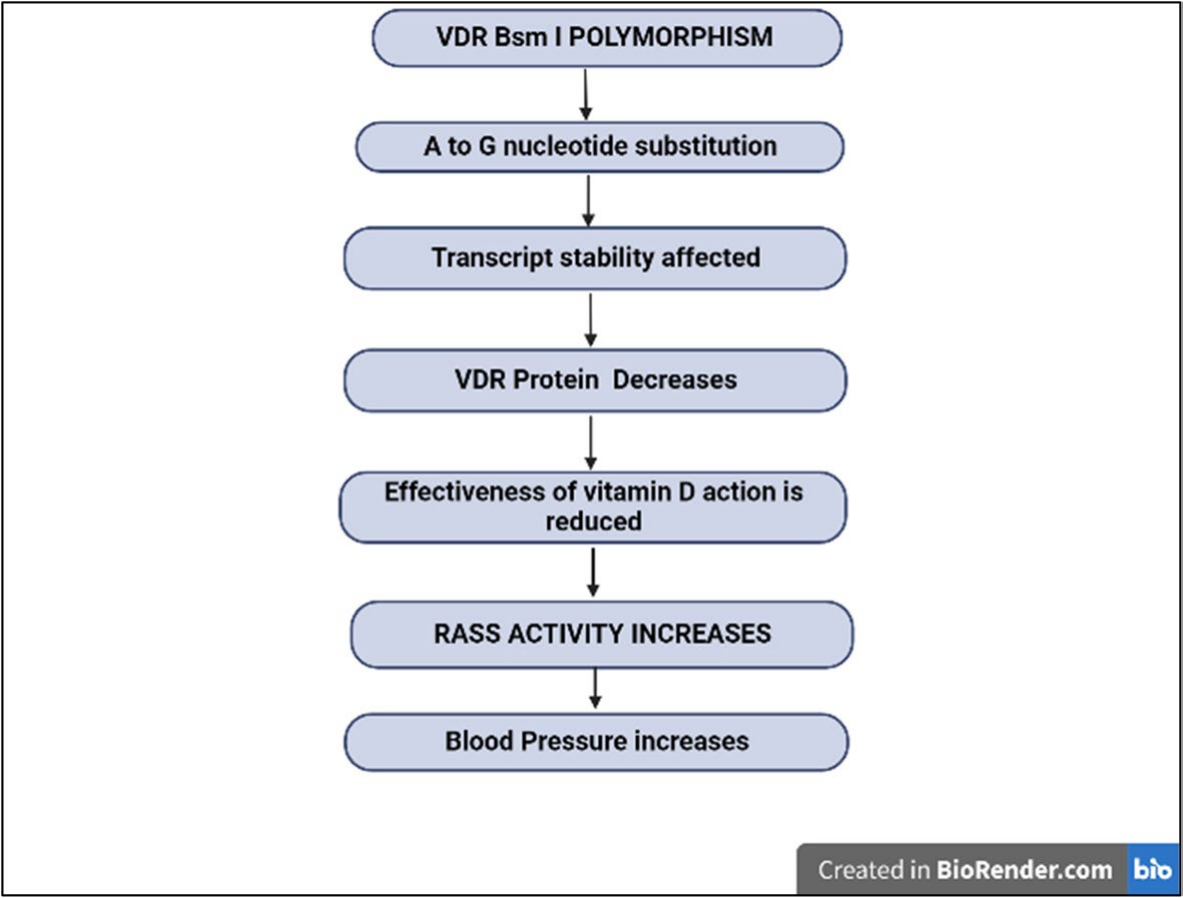
Proposed mechanism of vitamin D receptor (VDR; *Bsm I*) polymorphism in susceptibility to essential hypertension

*Bsm I* is a nucleotide substitution from A to G found in intron 8 that affects transcript stability. It is in linkage disequilibrium with other polymorphisms, and its association with certain diseases is most likely the result of this phenomenon (Fig. 5) [60].

A previous GWAS reported that the SNP *Bsm I* (rs1544410) in the *VDR* gene is associated with hypertension in Spanish population, systolic BP (SBP) with *Bsm I* (rs1544410) CC genotype was higher than TC or TT genotypes in men but not in women [61]. On the contrary, in a Korean study, *Bsm I* T allele carriers had higher SBP, higher diastolic BP (DBP), and higher prevalence of hypertension than CC carriers [62]. Recently, a prospective cohort of American men found suggestive evidence for associations of VDR *Bsm I* and *Fok I* polymorphisms with hypertension risk [32].

Regarding the role of SNP *Bsm I* in other diseases, Abouzid et al. [38] analyzed its association with CVDs and reported that GA *Bsm I* genotypes had an increased risk of obesity and also found that AA *Bsm I* genotype can be protective against CVD. Recently, Nugroho et al. [63] reported no association of this SNP with diabetic kidney disease. However, Nam et al. [37] reported that *Bsm I* polymorphism *VDR* gene were associated with obesity in Korean patients with type 2 diabetes mellitus. Further Liu et al. [34] found that *VDR Bsm I* (rs1544410) polymorphism was associated with gestational diabetes mellitus in Asian and African population [34].

This review identified and analyzed studies that investigated the relationship of *VDR* (*Fok I* and *Bsm I*) polymorphisms with essential hypertension. Swapna et al. [31] examined the relationship between *Fok I* polymorphism and essential hypertension in South Indian population. A total of 480 subjects were included in the study, with 280 hypertensive subjects and 200 healthy controls ranging in age from 35 to 60 years. They observed a significant association of *Fok I* polymorphism with essential hypertension. They also observed a significant difference in the frequencies of genotypes and alleles at the *VDR* locus. The FF, ff, Ff exhibited frequencies of 53.6, 10.7, and 35.7%, respectively, in hypertensive individuals, and 34, 15, and 51%, respectively, in healthy normotensive subjects. The risk of hypertension was calculated from the odds ratio (OR). Notably, men and women with the FF genotype had a higher risk of hypertension (men: OR, 2.020; 95% confidence interval [CI], 1.228–3.322; women: OR, 2.467; 95% CI, 1.246–4.4881) and in individuals with a positive family history of hypertension (OR, 2.011; 95% CI, 1.119–3.616), smoking (OR, 3.686; 95% CI, 1.414–9.611), and alcohol consumption (OR, 2.239; 95% CI, 0.983–5.096) [31].

In accordance with the results of previous study, another study was undertaken by Prasad et al. [13] in southeast population of India to determine the status of *Fok I VDR* gene polymorphism along with vitamin D levels and BP in patients with essential hypertension. A total of 400 subjects were included in the study, with 200 hypertensive subjects (SBP ≥140 mmHg and DBP ≥90 mmHg) and 200 healthy controls ranging in age from 25 to 60 years. They observed a significant association of SBP with vitamin D levels. A reduced level of vitamin D was observed in essential hypertension patients. Risk of hypertension was calculated from ORs. The ff genotype was found to be 8.06 times more likely to have essential hypertension than FF genotypes (OR, 8.06; 95% CI, 3.71–17.47; *P* = 0.0001).

Glocke et al. [64] conducted a study to compare the prevalence of *Fok I* polymorphism in 101 elderly (> 90 years) and 208 young subjects (< 90 years) of both sexes (Caucasians of German descent). A negative effect of *Fok I* polymorphism on DBP was observed in the elderly group (*P* < 0.05), especially in those with the ff genotype because they had a lower mean BP of 70 ± 2.12 mmHg than those with FF genotype (82.9 ± 3.10 mmHg) and Ff genotype (76.17 ± 2.69 mmHg) [64]. The prevalence of hypertension was lower in subjects with ff and Ff genotypes than in those with FF genotype; however, the difference was not statistically significant. The lifespan of the subjects can also be affected by this polymorphism [13].

Wang et al. [32] conducted a prospective study in 1211 Caucasian American men with a minimum and maximum follow-up period of 15.2 and 27.4 years, respectively. In this study, 695 subjects were diagnosed with hypertension. The prevalence of *Fok I* and *Bsm I* polymorphism was investigated in 885 subjects and 998 subjects, respectively, and the majority of hypertensive patients had a polymorphism of the *VDR* gene. The association of *Fok I* polymorphism with the risk for hypertension was found only in the recessive model. The ff genotype in model 2 had a multivariate hazard ratio (HR) of 1.32 (95% CI, 1.03–1.70) for the incidence of hypertension. The correlation between 25(OH)D level and the risk for hypertension was higher in subjects with the ff genotype than in those with the Ff and FF genotypes. They also observed bB or BB genotype of the *VDR Bsm I* polymorphism were linked to an increased risk of hypertension. The bB and BB genotype in model 2 had a multivariable HR of 1.27 (95% CI, 1.04–1.55) and 1.19 (95% CI, 0.90–1.56), respectively, for incidence of hypertension. Combined bB and BB genotypes, the HR was 1.25 (95% CI, 1.04–1.51) found only in the recessive model. There was no significant interaction between circulating vitamin D metabolites and *VDR* gene polymorphisms in relation to hypertension risk [32].

Cottone et al. [60] conducted a study in 71 patients with essential hypertension and 72 control subjects of both sexes, aged 18 to 75 years, in Italy. The frequencies of FF, Ff, and ff genotypes in hypertensive patients were 50.7, 42.3, and 7.0%, respectively, and in control subjects were 40.3, 50.0, and 9.7%, respectively. The allelic frequencies of F and f were 71.8 and 28.2%, respectively, in hypertensive patients, and 65.3 and 34.7%, respectively, in normotensive individuals. The DBP was different for all three genotypes of *Fok I* polymorphism (*P* = 0.018). Patients with the ff genotype had a higher DBP than those with the Ff genotype (*P* = 0.002). A negative correlation was observed between 25(OH)D levels and pulse BP, and this correlation was statistically significant in patients with the Ff genotype (r = − 0.474, *P* = 0.035) and between 25(OH)D and 24-hour SBP in patients with Bb *Bsm I* genotypes (r = − 0.397, *P* = 0.020). The frequencies of BB, bb, and Bb genotypes in hypertensive patients were 22.5, 57.8, and 19.7%, respectively, and 20.8, 52.8, and 26.4%, respectively, in control subjects. In hypertensive patients, the allelic frequencies of B and b were 51.5 and 48.5%, respectively, and 47.2 and 52.8%, respectively, in normotensive individuals. There was no link found between a particular genotype or allele and hypertension. A link between polymorphism and plasma renin activity was also not found [60].

In another study, Errouagui et al. [65] examined 177 hypertensive (aged 45.47 to 68.41 years) and 222 normotensive subjects (aged 34.67 to 64.59 years) of both sexes in Morocco, and found a strong association between *Fok I* polymorphism and hypertension in codominant, dominant, and recessive genetic models, whereas no association was observed between *Bsm I* polymorphism and hypertension in all genetic models. The ff genotype was significantly less common in hypertensive patients than in controls (OR, 0.24; 95% CI, 0.10–0.58; *P* = 0.002). The average vitamin D concentrations in subjects with the FF, Ff, and ff genotypes were 28.06 ± 10.57, 29.04 ± 11.97, and 26.40 ± 19.15 ng/mL, respectively. However, the differences in vitamin D concentrations between subjects with FF and Ff were not statistically significant [65].

Jia et al. [66] studied 2409 hypertensive and 3063 normotensive people from a community-based epidemiological survey in Jiangsu Province, China. The majority of the population was Han. After controlling for confounding factors (sex, age, body mass index, total cholesterol, triglycerides, high-density lipoprotein and low-density lipoprotein cholesterol, and smoking), the correlation of *Fok I* polymorphism with lower risk of hypertension in men was significant. For the additive, dominant, and recessive models, the ORs (95% CI) were 0.828 (0.74–0.927, *P* = 0.001), 0.75 (0.631–0.89, P = 0.001), and 0.816 (0.67–0.995, *P* = 0.044), respectively. The Ff/ff genotype was associated with lower BP than the FF genotype (*P* = 0.002) [66].

Gussago et al. [67] conducted another study on 70-year-old subjects and centenarians of both sexes in northern Italy. The frequencies of the FF, Ff, and ff genotypes in centenarians were 47.4, 42.1, and 10.5%, respectively, with F being the most common allele (68.4%). In the control group, the frequencies of the FF, Ff, and ff genotypes were 48.4, 38.7, and 12.9%, respectively, with F being the most common allele (67.8%). Furthermore, the FF genotype was associated with a higher prevalence of hypertension than the Ff and ff genotypes (*P* = 0.015) [67].

A meta-analysis conducted by Nunes et al. [47] in Brazil reported a significant association of *Fok I* polymorphism with hypertension. F and f allele frequencies were 55.9 and 44.1% in hypertensive patients, respectively, and 54.3 and 44.7% in control subjects [47]. However, a meta-analysis conducted in Chinese population by Zhu et al. [43] found no correlation of *Fok I* polymorphism with susceptibility to hypertension. They also observed that *Bsm I* polymorphism was associated with susceptibility to hypertension. The frequency of *VDR Bsm I* AA genotype decreased in hypertension patients compared with healthy controls. The population carrying *VDR Bsm I* AA genotype (OR, 0.69; 95% CI, 0.54–0.89; *P* = 0.005) had lower susceptibility to hypertension relative to those carrying GA or GG genotype (OR, 1.32; 95% CI, 1.05–1.68; *P* = 0.02). The frequency of A allele was higher in the case group than that of control group (OR, 0.83; 95% CI, 0.69–0.99; *P* = 0.04) [43]. In accordance with findings in Chinese population, a preliminary study conducted by Abouzid et al. [38] reported *Fok I* TT genotype was associated with a higher incidence of hypertension whereas they did not observe a significant association between hypertension and *Bsm I* polymorphism.

Both of these polymorphisms have been linked to an increased risk of essential hypertension with conflicting results [13, 32, 68]. The *Fok I* polymorphism affects the length and function of the *VDR* protein, resulting in truncated protein formation. The inactivation of *VDR* is caused by a lack or excess of vitamin D and the inactivation of its biologically active form. *Bsm I* is a nucleotide substitution from A to G in intron 8 that affects transcript stability. It is in linkage disequilibrium with other polymorphisms, and its association with certain diseases is most likely due to this phenomenon. The findings of the studies support the hypothesis that *VDR (Fok I* and *Bsm I)* polymorphisms may be associated with susceptibility to essential hypertension. Furthermore, the age and ethnicity of an individual are important factors to consider when studying hypertension. High BP can be caused by the inhibition of renin activity and the subsequent increase in levels of angiotensin II, a potent vasoconstrictor. Surprisingly, 1,25(OH)_2_D_3_ levels influences renin expression, resulting in hypertension. However, the number of studies in this area is still limited. Understanding the molecular and functional consequences of *VDR* polymorphisms is crucial for fully appreciating their significance and understanding their potential clinical implications.

The most notable mechanism linking vitamin D to hypertension is its role as a negative regulator of the RAAS. A meta-analysis study has indicated *Fok I* polymorphism of the VDR gene results in formation of a truncated protein that evidently increases the synthesis of renin and angiotensin II, thereby promoting the development of hypertension [47]. Enhanced understanding of the genetic differences in hypertensive population may be a further step in developing individualized treatment strategy. More so, it is worth investigating if direct renin inhibitors e.g., Aliskerin (presently second line of drugs) may be better suited and effective in this set of patients.

Over and above, as genetic technology becomes increasingly inexpensive and accessible, *Fok I* and *Bsm I* genotyping can be used as a screening tool in at risk population to assess future risk of developing hypertension.

## Conclusion

In India, hypertension has consistently been one of the leading causes of morbidity and mortality. Many studies have linked *VDR* gene polymorphism with essential however some studies have focused on the triangular relationship of vitamin D, *VDR* polymorphism, and essential hypertension, but the results of these studies were conflicting. As these studies have certain limitations, such as the fact that the individuals included in the study are of various age and ethnicities. These studies do not yield consistent findings regarding the risk of hypertension and its association with the frequency of *Fok I* and *Bsm I* genotypes. More research with larger samples is needed after removing confounding factors to determine whether VDR gene polymorphism is protective or a risk factor for the development of essential hypertension.

## Data Availability

All data generated or analyzed during this study are included in this published article.

## Abbreviations

1,25(OH)_2_D_3_: 1,25-Dihydroxyvitamin D
25(OH)D: 25-Hydroxyvitamin D
AC: Adenylate cyclase
BP: Blood pressure
CAD: Coronary artery disease
cAMP: Cyclic adenosine monophosphate
CBP: Cyclic adenosine monophosphate binding protein
CI: Confidence interval
CRE: Cyclic adenosine monophosphate response element
CREB: Cyclic adenosine monophosphate dependent response element binding protein
CREM: Cyclic adenosine monophosphate response element modulator
CVD: Cardiovascular disease
DBP: Diastolic blood pressure
DM: Diabetes mellitus
Gα_s_: G_s_ protein α subunit
GDM: Gestational diabetes mellitus
GH: Gestational hypertension
GWAS: Genome-wide association studies
HR: Hazard ratio
mRNA: Messenger RNA
OHase: Hydroxylase
OR: Odds ratio
P: Phosphate
PKA: Protein kinase A
Pol: Polymerase
RA: Rheumatoid arthritis
RAAS: Renin-angiotensin-aldosterone system
RXR: Retinoid X receptor
SBP: Systolic blood pressure
SNP: Single nucleotide polymorphism
UTR: untranslated region
VDR: Vitamin D receptor
